# Audit of section 2 and section 3 mental health act paperwork in Derby inpatient psychiatric units using an audit tool by Mason et al. (2012)

**DOI:** 10.1192/bjo.2021.294

**Published:** 2021-06-18

**Authors:** Abigail Pearson, Andrew Horton, Mike Akroyd

**Affiliations:** 1 Former Clinical Leadership Fellow at Derbyshire Healthcare NHS Foundation Trust, CT1 Doctor at Sheffield Health and Social Care; 2 Consultant Psychiatrist, Derbyshire Healthcare NHS Foundation Trust

## Abstract

**Aims:**

Complete an audit of Section 2 and Section 3 Mental Health Act Paperwork in Derby Inpatient psychiatric units using an audit tool developed in a study by Mason et al. (2012).

**Background:**

The 1983 Mental Health Act enables doctors approved on behalf of the Secretary of State under Section 12 to be able to make recommendations for the detention of individuals with a mental health problem where the degree and/or nature, and associated risk to that person's health or safety, or that of others, makes inpatient care necessary. For the detention and the associated deprivation of their liberty to be lawful, it is necessary that the clinical situation meets certain criteria as outlined in the Mental Health Act.

**Method:**

Ward status was reviewed for each inpatient ward in Derby and the first five patients alphabetically, who were detained under sections 2 or 3 were selected. The Mental Health Act medical recommendation documents were reviewed according to the necessary criteria, using an assessment tool generated from a study by Mason et al. in 2012 ‘Compulsion under the Mental Health Act 1983: audit of the quality of medical recommendations’. A junior colleague was trained to analyse Mental Health Act paperwork using the audit tool. Medical recommendations were reviewed and rated as ‘clear’, ‘implied’ or ‘none’ for each criterion.

**Result:**

Evidence of a mental health problem and the nature or degree of illness was well documented. Evidence regarding why informal admission was not appropriate was also reasonable but with room for improvement. Poor compliance was evident mostly in relation to the justification related to risk to health, safety or others, the lowest clearly documented percentage of these appear to be regarding health.

**Conclusion:**

From analysing the documentation, often written justification incorporated general safety as a whole; however health and safety are identified by the mental health act as separate criterion requiring clear justification of each. In a number of occasions people failed to identify which of the three risk categories were relevant for the patient. Potential criticisms of this audit include the subjective nature of the interpretation of clearly explained and implied and that data analysis was completed by a non-section 12 approved doctor. Data were presented at the local weekly academic teaching to raise awareness of the results and a recommendation was made for the subject to be included in the junior doctor induction.

